# Dexmedetomidine versus Magnesium Sulfate as Adjunct during Anesthesia for Laparoscopic Colectomy

**DOI:** 10.1155/2016/7172920

**Published:** 2016-03-09

**Authors:** Pierre Zarif, Ahmed Abdelaal Ahmed Mahmoud, Mohamed Mohamed Abdelhaq, Hany M. S. Mikhail, Ahmed Farag

**Affiliations:** ^1^Department of Anesthesiology, Faculty of Medicine, Cairo University, Giza, Egypt; ^2^Department of Anesthesiology, Faculty of Medicine, Beni-Suef University, Beni Suef, Egypt; ^3^Department of General Surgery, Faculty of Medicine, Cairo University, Giza, Egypt

## Abstract

*Objectives*. To compare dexmedetomidine versus magnesium during laparoscopic colectomy.* Patients and Methods*. 51 patients were randomly allocated into 3 groups: group C (control) received saline infusion, group D dexmedetomidine 1 g/kg and then 0.4 g/kg/hr, and group M MgSO_4_ 2 g and then 15 g/kg/min. Intraoperative hemodynamics were measured before and 1 min after intubation (*T*1 and* T*2), before and 5 min after peritoneal insufflation (*T*3 and* T*4), before and 5 min after 30° Trendelenburg position (*T*5 and* T*6), 5 min after resuming flat position (*T*7), 5 min after peritoneal deflations (*T*8), after extubation (*T*9), and at time of admission to PACU (*T*10). Recovery time and degree of sedation were assessed.* Results*. HR and MAP were significantly higher in* T*2,* T*4, and* T*6 compared to* T*1,* T*3, and* T*5, respectively, in all groups with lower measurements in groups D and M compared to group C. Mean of collective measurements was significantly higher in group C. Recovery time and sedation score were significantly higher in groups D and M. Time to Aldrete score of ≥9 was significantly longer in groups D and M.* Conclusion*. Both drugs ameliorate the pressor responses during LC with a nonsignificant difference. This study is registered with PACTR201602001481308.

## 1. Introduction

Nowadays laparoscopic surgery is going to be the first choice for surgical management of various indications, especially with the well-trained laparoscopic surgeon. The benefits of minimal access techniques include less pain, early mobilization, shorter hospital stay, and better cosmetic results, which have further increased its applications [1, 2].

During general anesthesia laryngoscopy, tracheal intubation and extubation are the critical events provoking transient but marked sympathoadrenal response manifesting by hypertension and tachycardia. In addition, in laparoscopic surgery, CO_2_ is routinely used to create pneumoperitoneum, which causes increased plasma level of catecholamine and vasopressin. Elevation of intra-abdominal pressure with raised diaphragm causes various adverse effects on the cardiovascular system such as decreased cardiac output, elevated arterial pressure, and increased systemic and pulmonary vascular resistance leading to hypertension and tachycardia [3, 4].

Severe increases in arterial pressure can be a risk factor in patients with preexisting hypertension, ischaemic heart disease, or increased intracranial pressure. Opioids, alpha-2-adrenergic agonists, beta-blocking agents, or vasodilators are often used to avoid circulatory response to pneumoperitoneum [5].

Magnesium is well known to block the release of catecholamines from both adrenergic nerve terminals and the adrenal gland, and intravenous magnesium sulfate inhibits catecholamine release associated with laryngoscopy. Moreover, magnesium produces vasodilator effect by acting directly on blood vessels, and high-dose magnesium attenuates vasopressin-stimulated vasoconstriction [6, 7].

On the other hand, dexmedetomidine is an alpha-2-adrenergic agonist; it has properties of analgesia, sympatholytic effect, and sedation without respiratory depression. It decreases opioid requirements and stress response to surgery ensuring a stable hemodynamic state. Its distribution half-life is around six min, so it can be used to attenuate the stress response to laryngoscopy [8–10].

Our study aimed at assessment of hemodynamic changes during laparoscopic colectomy under pneumoperitoneum in 30° Trendelenburg position and the impact of intraoperative dexmedetomidine and magnesium sulfate infusions on these changes.

## 2. Patients and Methods

The study was carried out at Cairo University Hospital since Jan 2010 till Aug 2013. After approval of the study protocol by the Local Ethical Committee and obtaining patients' written fully informed consents, all patients assigned for laparoscopic colorectal resection were enrolled in the study. Patients who required preoperative neoadjuvant therapy, are generally unfit for resection surgery, or had inoperable lesion requiring relieving or dysfunctioning colostomy were excluded from the study. Patients with cardiopulmonary diseases, renal or liver impairment, or allergy to any of the used drugs were also excluded from the study. Morbidly obese with body mass index (BMI) >35 kg/m^2^ was not enrolled in the study.

Patients were randomly, using sealed envelopes, allocated to 3 groups: group C assigned to receive saline infusion as placebo, group D assigned to receive dexmedetomidine infusion, and group M assigned to receive intravenous infusion of magnesium sulfate.

Fifteen minutes before surgery, all patients were premedicated with midazolam 1-2 mg and given drugs as follows: group C: saline infusion 20 mL over 15 min; group D: dexmedetomidine loading 1 mic/kg in 20 mL normal saline over 15 min; and Group M: magnesium sulfate loading 2 g, 20 mL over 15 min. Baseline data (*T*0) including systolic blood pressure (SBP), diastolic blood pressure (DBP), mean arterial blood pressure (MAP), and heart rate (HR) were noninvasively determined and monitored and then anesthesia was conducted with fentanyl 1 mic/kg and propofol 2 mg/kg, followed by cisatracurium (0.15–0.2 mg/kg) to facilitate tracheal intubation, and then maintenance of loaded drugs was continued as follow: Group C: saline infusion as placebo; Group D: dexmedetomidine infusion at rate of 0.4 *μ*g/kg/hr; and Group M: magnesium sulfate infusion at rate of 15 *μ*g/kg/min.

Controlled ventilation was adjusted to an end tidal CO_2_ concentration of 30–35 mmHg and to ensure SpO_2_ of 97%. Anesthesia was maintained in all groups by sevoflurane 2 MAC with 50% oxygen and 50% air and maintenance doses of cisatracurium. All groups received ketorolac 30 mg IV slowly and paracetamol 1 g IV at the beginning of surgery and Ringer acetate solution was given at a rate of 10 mL/kg/hr during anesthesia. Infusions and sevoflurane inhalation were stopped just at the end of surgery, then atropine sulfate 0.02 mg/kg and neostigmine 0.04 mg/kg were administered IV for reversal of muscle relaxation, and the patient was extubated. Following extubation, patients were maintained on supplemental O_2_ until being awake in the recovery room. Any pain in the recovery room was managed by pethidine 1 mg/kg IV if needed. The recovery time was estimated in all groups as the time elapsed since stoppage of sevoflurane and infusions, till the time the patient achieves a modified Aldrete scoring of ≥9. The degree of sedation was assessed at the end of end of surgery and 30 and 120 minutes thereafter using the Brussels Sedation Scale (BSS), [11] where 1 = sedated and unarousable, 2 = sedated but responding to painful not auditory stimuli, 3 = sedated but responding to auditory stimuli, 4 = awake and calm, and 5 = agitated.

Intraoperative hemodynamic measures were determined before intubation (*T*1), 1 min after intubation (*T*2), before and 5 min after peritoneal insufflation (*T*3  and  *T*4), before and 5 min after tilting the table to 30° Trendelenburg position (*T*5  and *T*6), 5 min after resuming flat position (*T*7), 5 min after peritoneal deflation (*T*8), after extubation (*T*9), and at time of admission to PACU (*T*10). Mean of collective intraoperative changes of HR, SBP, DBP, and MAP was calculated collectively for measurements *T*1–*T*9.

## 3. Statistical Analysis and Sample Size Calculation

Sample power was calculated according to Kraemer and Theimann [12] using their proposed figure. Considering that laparoscopic colectomy is the unusual procedure for resection of colorectal cancer and that the hemodynamic changes during laparoscopic surgery under pneumoperitoneum are a frequent event and also considering a standard nomogram [13], we determined a sample size of 17 patients per group to obtain a study power of 80% to detect a difference at the significance level of 5%.

Collected data was presented as mean (±SD), numbers, and percentages, as appropriate. Categorical variables were analyzed using chi-square (*χ* ^2^) test. Continuous variables were analyzed using unpaired Student's *t*-test or univariate two-group repeated measures “mixed-design” analysis of variance (ANOVA) with post hoc Dunnett's test as appropriate. Nominal and nonnormally distributed variables were analyzed using Mann-Whitney *U* test. Statistical analysis was done by using the computer program SPSS (Statistical Package for Social Sciences), Version 20, 2011. *P* value < 0.05 was considered statistically significant.

## 4. Results

The study included 51 patients, 36 males and 15 females, with mean age of 61.2 ± 7.3, range: 45–68 years. There were eight patients of normal weight, 25 patients were overweight, and 18 patients were obese with a total mean BMI of 29.3 ± 3.1, range: 22.6–34.1 kg/m^2^. Details of patients' demographic data are shown in Table 1. There was a nonsignificant (*P* > 0.05) difference between studied groups with regard to demographic data.

Throughout the duration of surgery, HR showed variability during times of stress with significantly (*P* < 0.05) higher HR records at *T*2, *T*4, and *T*6 compared to that recorded at *T*1, *T*3, and *T*5, respectively, in all studied patients. The ameliorative effect of studied drugs was evident and manifested as higher significance of differences between these measures in group C than in groups D and M. Concerning the HR records throughout operative time till PACU transfer, HR records were significantly lower in groups D and M compared to group C at all times of recording with nonsignificantly (*P* > 0.05) lower HR records in group D compared to group M (Table 2).

Throughout the duration of surgery, MAP showed variability during times of stress with significantly (*P* < 0.05) higher MAP measurements at *T*2, *T*4, and *T*6 compared to that measured at *T*1, *T*3, and *T*5, respectively, in all studied patients. However, both studied drugs provided better blood pressure stabilization as manifested by the lower significance of differences between these measures in groups D and M than in group C. MAP measurements determined throughout operative time till PACU transfer were significantly lower in groups D and M compared to group C at all times of recording with nonsignificant (*P* > 0.05) difference in favor of group D (Table 3).

Mean of collective HR records from *T*1 to *T*9 was significantly (*P* < 0.05) higher in group C compared to groups D and M with significantly (*P* < 0.05) lower HR records in group D compared to group M. Mean of collective blood pressure measurements since *T*1 to *T*9 was significantly (*P* < 0.05) higher in group C compared to groups D and M with nonsignificantly (*P* > 0.05) lower measurements in group D compared to group M (Table 4).

Studied patients showed nonsignificant (*P* > 0.05) difference concerning operative and surgical postoperative data (Table 5). Both studied drugs significantly prolonged postanesthetic recovery with significantly (*P* < 0.05) higher frequency of sedated patients at the end of surgery and 15 and 30 minutes thereafter compared to group C. However, at 15 and 30 minutes after surgery, the frequency of awaked patients showed nonsignificant (*P* > 0.05) difference between the three groups, but the frequency of agitated patients was significantly (*P* < 0.05) higher in group C compared to other groups. Mean time till reaching modified Aldrete score of ≥9 was significantly (*P* < 0.05) longer in groups D and M compared to group C with a nonsignificant (*P* > 0.05) difference between both groups (Table 6).

## 5. Discussion

The key element in laparoscopic surgery is the creation of pneumoperitoneum and the physiologic effect CO_2_. Moreover, pelvic and lower abdominal laparoscopic surgeries have a special concern wherein the body must be positioned in Trendelenburg position with leg elevation up to 30° to give room for the surgeon to deal with pelvic organs. This positioning imposed more hemodynamic burden secondary to the more diaphragmatic splinting action and increased venous return [14, 15].

In this study, the patients were assigned for laparoscopic colorectal resection so the body must be positioned in Trendelenburg position. The results showed that HR and MAP measurements were significantly higher in *T*2, *T*4, and *T*6 compared to *T*1, *T*3, and *T*5, respectively, in all groups with lower measurements in groups D and M compared to group C. Also, the mean of collective HR records was significantly higher in group C compared to groups D and M (*P* < 0.05), with significantly (*P* < 0.05) lower HR records in group D compared to group M. Mean of collective blood pressure measurements was significantly (*P* < 0.05) higher in group C compared to groups D and M with nonsignificantly (*P* > 0.05) lower measurements in group D compared to group M. Studied patients also showed that both studied drugs significantly prolonged postanesthetic recovery compared to group C (*P* < 0.05). However, at 15 and 30 minutes after surgery, the frequency of awaked patients showed nonsignificant difference between the three groups. But the frequency of agitated patients was significantly higher in group C compared to other groups. Mean time till reaching modified Aldrete score of ≥9 was significantly (*P* < 0.05) longer in groups D and M compared to group C with a nonsignificant (*P* > 0.05) difference between both groups.

The results of this study about effect of magnesium sulfate on hemodynamics intraoperative correlate well with Lee and Kwon [16], who found that preoperative intravenous magnesium sulfate attenuated arterial pressure increases during the predelivery period and was recommended as an adjuvant during general anesthesia for caesarean section to avoid perioperative pressor response resulting from inadequate anesthesia, analgesia, or both of them [16]. Jee et al. [5] also found that intravenous magnesium sulfate before pneumoperitoneum attenuates arterial pressure increases during laparoscopic cholecystectomy and this attenuation is apparently related to reductions in the release of catecholamine, vasopressin, or both of them [5]. Kalra et al. [17] assessed which of magnesium or clonidine attenuates hemodynamic stress response to pneumoperitoneum better and found that systolic blood pressure was significantly higher in control group as compared to study groups during pneumoperitoneum with no significant difference between magnesium and clonidine given in dose of 1 *μ*g/kg. However, the difference was better in patients receiving clonidine in dose of 1.5 *μ*g/kg [17].

The effect of magnesium on hemodynamics due to interact in the activation of membrane Ca-ATPase and Na-K-ATPase is involved in transmembrane ion exchanges during depolarization and repolarization phases, thus acting as a cell membrane stabilizer and also as an intracytoplasmic organelles stabilizer [7]. This calcium inhibitory effect of Mg causes central arteriolar vasodilatation and acts against vasospasm. Another mechanism could involve the reduction of catecholamine release with sympathetic stimulation, thereby decreasing the stress response to surgery. The analgesic effect of magnesium due to it blocks N-methyl-D-aspartate (NMDA) receptor which plays a significant role in the mechanisms underlying central sensitization in the spinal cord and is crucial for the establishment of several pain states [6, 7].

On the other hand, the results of this study about effect of dexmedetomidine on hemodynamics intraoperative correlate well with Kato et al. [18] who found that dexmedetomidine weakens arterial pressure preservation and HR responses after thigh cuff deflation, suggesting attenuated cardiovascular reflexes [18]. Klinger et al. [19] assessed the hemodynamic impact of dexmedetomidine administration in a large cohort of patients undergoing routine noncardiac surgery and found that a significantly higher percentage of patients in dexmedetomidine group met the composite endpoint criteria with no significant difference in the overall incidence of intraoperative hypotension and concluded that dexmedetomidine administration was associated with hemodynamic stability with lowering of hemodynamic stress response without more hypotension or bradycardia [19].

Concerning laparoscopic surgery, the obtained results supported what was previously reported by Smania et al. [20] who found that dexmedetomidine efficiently blocks the hemodynamic responses to nociceptive stimuli when combined with inhaled isoflurane for anesthesia of children submitted to laparoscopic video appendectomy [20].

The effect of dexmedetomidine on hemodynamics is due to decrease of sympathetic outflow from the locus coeruleus. Its sympatholytic effect leads to decrease of mean arterial blood pressure (MAP) and heart rate (HR) by reducing norepinephrine release. Its analgesic actions are mediated by releasing of substance P from the dorsal horn of the spinal cord [8].

As a further support for efficacy of both drugs, Bryskin and Weldon [21] used a combination of dexmedetomidine and magnesium sulfate for hemodynamic control during laparoscopic resection of pheochromocytoma and reported that cardiovascular stability was achieved [21].

Unfortunately, there was no comparative study between the hemodynamic effects of dexmedetomidine and magnesium sulfate during laparoscopic surgery; however, other comparative studies provided evidence for their superiority to other drugs used for the same target, where Salman et al. [22] compared dexmedetomidine with remifentanil in desflurane-based ambulatory gynecologic laparoscopic surgery and demonstrated that dexmedetomidine infusion causes a relatively slow recovery with reduced postoperative nausea, vomiting, and analgesic requirements, and similar hemodynamics compared to remifentanil, and may be an alternative to remifentanil in ambulatory anesthesia [22].

## 6. Conclusion

Intraoperative infusion of either dexmedetomidine or magnesium sulfate could ameliorate the pressor responses to anesthetic and surgical manipulations during laparoscopic colectomy under pneumoperitoneum in 30° Trendelenburg position.

## Figures and Tables

**Table 1:** Patients' demographic data.

		Group C	Group D	Group M	*P* value
Age (years)	Strata				
	<50	1 (5.9%)	3 (17.6%)	2 (11.8%)	*P*_1_ = 0.067
	50–60	3 (17.6%)	3 (17.6%)	4 (23.5%)	*P*_2_ = 0.086
	>60	13 (76.5%)	11 (64.7%)	11 (64.7%)	*P*_3_ = 0.421
	Total	62.5 ± 4.9	60.9 ± 7.1	60.3 ± 7.3	*P*_1_ = 0.156
					*P*_2_ = 0.193
					*P*_3_ = 0.791

Gender	Males Females	11 (64.7%) 6 (35.3%)	13 (76.5%) 4 (23.5%)	12 (70.6%) 5 (29.4%)	*P*_1_ = 0.086 *P*_2_ = 0.106 *P*_3_ = 0.519

BMI data	Weight	83.4 ± 6.4	84.5 ± 7.5	84.6 ± 8	*P*_1_ = 0.482
					*P*_2_ = 0.307
					*P*_3_ = 0.619
	Height	169.2 ± 4.2	169.7 ± 4.3	169.6 ± 4	*P*_1_ = 0.308
					*P*_2_ = 0.295
					*P*_3_ = 0.456
	Strata				
	Normal	3 (17.6%)	2 (11.8%)	3 (17.6%)	*P*_1_ = 0.071
	Overweight	7 (41.2%)	10 (58.8%)	8 (47.1%)	*P*_2_ = 0.532
	Obese	7 (41.2%)	5 (29.4%)	6 (35.3%)	*P*_3_ = 0.102
	Total	29.2 ± 2.8	29.4 ± 3.3	29.5 ± 3.4	*P*_1_ = 0.285
					*P*_2_ = 0.231
					*P*_3_ = 0.495

ASA	Grade I	11 (64.7%)	12 (70.6%)	13 (76.5%)	*P*_1_ = 0.077
	Grade II	4 (23.5%)	2 (11.8%)	3 (17.6%)	*P*_2_ = 0.059
	Grade III	2 (11.8%)	3 (17.6%)	1 (5.9%)	*P*_3_ = 0.119

Data are presented as mean ± SD and numbers; percentages are in parenthesis. BMI: body mass index; *P*_1_: significance of difference between groups C and D; *P*_2_: significance of difference between groups C and M; *P*_3_: significance of difference between groups D and M.

**Table 2:** Mean HR records determined throughout the operative time.

	*T*0	*T*1	*T*2	*T*3	*T*4	*T*5	*T*6	*T*7	*T*8	*T*9	*T*10
Group C	82.9 ± 4	66 ± 2.7	69.5 ± 3.8	70.8 ± 4	74.2 ± 3.6	71.8 ± 3	75 ± 5	71.1 ± 3.5	73.2 ± 4	74.7 ± 4.	76.9 ± 5.2
		*P* = 0.001		*P* = 0.001		*P* = 0.009					

Group D	83.4 ± 4.5	61.2 ± 2.9	65 ± 5.3	62.7 ± 3.8	66.8 ± 5.8	63.4 ± 4.8	67.1 ± 3.8	64.2 ± 2	64.8 ± 2.9	66.7 ± 3.8	71.8 ± 3.4
		*P* = 0.041		*P* = 0.011		*P* = 0.026					

Group M	83.7 ± 3.1	62.1 ± 2.1	66.8 ± 3.9	63.1 ± 3.9	67.1 ± 6.1	64.2 ± 4.9	69.2 ± 7.4	65.7 ± 5.5	65.8 ± 3.9	67.4 ± 5.5	72.4 ± 3
		*P* = 0.005		*P* = 0.029		*P* = 0.036					

Intergroup difference	*P*_1_	=0.001	=0.029	=0.0008	=0.001	0.0008	=0.001	=0.0008	=0.0006	=0.001	=0.031
	*P*_2_	=0.022	=0.112	=0.002	=0.001	=0.0008	=0.044	=0.004	=0.0006	=0.007	=0.003
	*P*_3_	=0.387	=0.255	=0.925	=0.859	=0.604	=0.379	=0.649	=0.475	=0.777	=0.534

*P*: significance between times identified; *P*_1_: significance of difference between groups C and D; *P*_2_: significance of difference between groups C and M; *P*_3_: significance of difference between groups D and M.

**Table 3:** Mean (±SD) MAP records determined throughout the operative time.

	*T*0	*T*1	*T*2	*T*3	*T*4	*T*5	*T*6	*T*7	*T*8	*T*9	*T*10
Group C	94.6 ± 3.7	73.9 ± 2.9	80.5 ± 2.8	75.1 ± 3	78.4 ± 2.6	74.9 ± 2.1	78.3 ± 3.5	75.9 ± 3.2	76.6 ± 3	79 ± 3.3	87.7 ± 4.1
		*P* = 0.0007		*P* = 0.003		*P* = 0.0009					

Group D	95.7 ± 3.8	66.8 ± 2.8	72.3 ± 3.7	68.2 ± 3.3	72.4 ± 4.6	67.6 ± 4.1	73.6 ± 2.7	72.7 ± 2.1	73.6 ± 2.5	75.4 ± 3.1	84.4 ± 2.7
		*P* = 0.001		*P* = 0.019		*P* = 0.001					

Group M	94.1 ± 3.8	68 ± 3.7	74.4 ± 2.6	69.6 ± 2.5	73.4 ± 4.8	69 ± 3.2	73.5 ± 5.1	72.7 ± 3.3	73.4 ± 2.7	76.4 ± 3.2	85.1 ± 3.5
		*P* = 0.0007		*P* = 0.002		*P* = 0.002					

Intergroup difference	*P*_1_	=0.001	=0.0009	=0.0009	=0.002	=0.0009	=0.002	=0.028	=0.036	=0.035	=0.029
	*P*_2_	=0.001	=0.001	=0.0007	=0.007	=0.0009	=0.017	=0.041	=0.033	=0.042	=0.047
	*P*_3_	=0.136	=0.112	=0.177	=0.445	=0.224	=0.756	=0.932	=0.813	=0.868	=0.435

*P*: significance between times identified; *P*_1_: significance of difference between groups C and D; *P*_2_: significance of difference between groups C and M; *P*_3_: significance of difference between groups D and M.

**Table 4:** Mean (±SD) of collective hemodynamic variable estimated from *T*1 to *T*9 period.

	Group C	Group D	Group M
HR (beats/min)	71.8 ± 2	64.7 ± 1.3	65.7 ± 1.8
		*P*_1_ = 0.0006	*P*_2_ = 0.0008
			*P*_3_ = 0.046

SBP (mmHg)	84.5 ± 1.7	80.2 ± 1.3	79.8 ± 1.8
		*P*_1_ = 0.0009	*P*_2_ = 0.0009
			*P*_3_ = 0.234

DBP (mmHg)	72.9 ± 2.2	67.1 ± 1.1	68.5 ± 2.3
		*P*_1_ = 0.0008	*P*_2_ = 0.0009
			*P*_3_ = 0.059

MAP (mmHg)	76.8 ± 1.6	71.5 ± 0.8	72.3 ± 1.4
		*P*_1_ = 0.0007	*P*_2_ = 0.0009
			*P*_3_ = 0.076

*P*_1_: significance of difference between groups C and D; *P*_2_: significance of difference between groups C and M; *P*_3_: significance of difference between groups D and M.

**Table 5:** Operative and immediate postoperative surgical data.

	Group C	Group D	Group M	*P* value
Operative time (min)	123.6 ± 15.7	129.4 ± 12.4	128.8 ± 23.8	*P*_1_ = 0.186 *P*_2_ = 0.569 *P*_3_ = 0.850

Amount of blood loss (mL)	309.1 ± 94.1	281.5 ± 50.3	295.9 ± 97.2	*P*_1_ = 0.480 *P*_2_ = 0.501 *P*_3_ = 0.687

Need for blood transfusion				
Yes	5 (29.4%)	3 (17.6%)	4 (23.5%)	*P*_1_ = 0.079 *P*_2_ = 0.236 *P*_3_ = 0.318
No	12 (70.6%)	14 (82.4%)	13 (76.5%)	

Time till 1st flatus passage (days)	1.8 ± 0.7	2 ± 0.7	1.7 ± 0.8	*P*_1_ = 0.448 *P*_2_ = 0.713 *P*_3_ = 0.332

Time till 1st oral fluid intake (days)	3 ± 0.8	2.6 ± 0.7	3.1 ± 0.9	*P*_1_ = 0.198 *P*_2_ = 0.480 *P*_3_ = 0.170

Time till 1st oral solid food (days)	3.1 ± 1	3.5 ± 0.6	3.3 ± 0.9	*P*_1_ = 0.083 *P*_2_ = 0.408 *P*_3_ = 0.396

Surgical complications				
Yes	3 (17.6%)	4 (23.5%)	2 (11.8%)	*P*_1_ = 0.467 *P*_2_ = 0.691 *P*_3_ = 0.106
No	14 (82.4%)	13 (76.5%)	15 (88.2%)	

PO hospital stay (days)	6.9 ± 1.2	7.1 ± 1.5	7 ± 1.6	*P*_1_ = 0.703 *P*_2_ = 0.908 *P*_3_ = 0.581

Data are presented as mean ± SD and numbers; percentages are in parenthesis. *P*_1_: significance of difference between groups C and D; *P*_2_: significance of difference between groups C and M; *P*_3_: significance of difference between groups D and M.

**Table 6:** Recovery data of studied patients.

	Group C	Group D	Group M	*P* value
*Brussels Sedation Scale (BSS)*				
At end of surgery				
I	5 (29.4%)	3 (17.6%)	2 (11.8%)	*P*_1_ = 0.069 *P*_2_ = 0.005 *P*_3_ = 0.575
II	12 (70.6%)	14 (82.4%)	15 (88.2%)	
15 min after end of surgery				
II	2 (11.8%)	5 (29.4%)	6 (35.3%)	*P*_1_ = 0.005 *P*_2_ = 0.005 *P*_3_ = 0.239
III	8 (47.1%)	9 (52.9%)	8 (47.1%)	
IV	7 (41.1%)	3 (17.7%)	3 (17.7%)	
30 min after end of surgery				
II	0	1 (5.9%)	2 (11.8%)	*P*_1_ = 0.006 *P*_2_ = 0.003 *P*_3_ = 0.093
III	2 (11.8%)	3 (17.7%)	4 (23.5%)	
IV	9 (52.9%)	10 (58.8%)	9 (52.9%)	
V	6 (35.3%)	3 (17.7%)	2 (11.8%)	

Time till reaching Aldrete score ≥9				
5 min	2 (11.8%)	0	0	*P*_1_ = 0.0009 *P*_2_ = 0.0008 *P*_3_ = 0.075
10 min	3 (17.6%)	1 (5.9%)	2 (11.8%)	
15 min	6 (35.3%)	2 (11.8%)	1 (5.9%)	
20 min	3 (17.6%)	4 (23.5%)	3 (17.6%)	
25 min	1 (5.9%)	6 (35.3%)	5 (29.4%)	
30 min	1 (5.9%)	3 (17.6%)	4 (23.5%)	
>30 min	1 (5.9%)	1 (5.9%)	2 (11.8%)	
Total time	16.5 ± 8.1	23.2 ± 6.4	24.1 ± 7.5	*P*_1_ = 0.0008 *P*_2_ = 0.0007 *P*_3_ = 0.180

Data are presented as mean ± SD and numbers; percentages are in parenthesis. *P*_1_: significance of difference between groups C and D; *P*_2_: significance of difference between groups C and M; *P*_3_: significance of difference between groups D and M.
